# Transient Biocompatible Polymeric Platforms for Long-Term Controlled Release of Therapeutic Proteins and Vaccines

**DOI:** 10.3390/ma9050321

**Published:** 2016-04-28

**Authors:** Handan Acar, Saikat Banerjee, Heliang Shi, Reihaneh Jamshidi, Nastaran Hashemi, Michael W. Cho, Reza Montazami

**Affiliations:** 1 Department of Mechanical Engineering, Iowa State University, Ames, IA 50011, USA; acarhandan@gmail.com (H.A.); reihaneh@iastate.edu (R.J.); nastaran@iastate.edu (N.H.); 2 Department of Biomedical Sciences, College of Veterinary Medicine, Iowa State University, Ames, IA 50011, USA; skt3187@iastate.edu (S.B.); shiyxtox@iastate.edu (H.S.); mcho@iastate.edu (M.W.C.); 3 Center of Advanced Host Defenses Immunobiotics and Translational Medicine, Iowa State University, Ames, IA 50011, USA; 4 Ames Laboratory, Department of Energy, Ames, IA 50011, USA

**Keywords:** transient platform, controlled release, polymer platform, long-term release

## Abstract

Polymer-based interpenetrating networks (IPNs) with controllable and programmable degradation and release kinetics enable unique opportunities for physisorption and controlled release of therapeutic proteins or vaccines while their chemical and structural integrities are conserved. This paper presents materials, a simple preparation method, and release kinetics of a series of long-term programmable, biocompatible, and biodegradable polymer-based IPN controlled release platforms. Release kinetics of the gp41 protein was controlled over a 30-day period via tuning and altering the chemical structure of the IPN platforms. Post-release analysis confirmed structural conservation of the gp41 protein throughout the process. Cell viability assay confirmed biocompatibility and non-cytotoxicity of the IPNs.

## 1. Introduction

Recent developments in pharmacology have triggered the emergence of therapeutic proteins for the treatment of human and animal diseases. These protein therapeutics include monoclonal antibodies, hormones, enzymes, growth factors, immunological molecules, and many more. The development of such protein therapeutics with diverse functions requires the design of new platforms that can meet the diverse delivery needs. However, administration of protein-based drugs can be challenging because of the chemical structure and sensitivity of proteins. The function of proteins is susceptible to environmental conditions, like pH and temperature. In the past, there have been substantial efforts to develop micro-actuators [1,2,3,4,5,6,7] for controlling miniaturized implantable containers that physically hold proteins and medicine; and release them through an actuator-controlled gate when needed [8,9]. Use of harsh conditions for platform formulation and protein encapsulation can denature the protein and reduce or abolish its biological activity upon release. Many protein-based drugs require frequent administrations to achieve high enough concentrations needed for biological activity in the body during long-term therapy. Platforms with long-term controlled-released mechanisms can ensure slow and sustainable release while protecting the protein from harsh conditions in the body [10].

The natural extracellular matrix of the tissue of living organisms can be taken as a model to develop delivery platforms. An extracellular matrix is an interpenetrating network (IPN) of cross-linked proteins interlaced with high molecular weight biomacromolecules [11]. IPN is a combination of two polymers in network form, at least one of which is synthesized and/or cross-linked in the immediate presence of the other [12,13]. Hydrogel IPNs, used as controlled-release systems, are capable of delivering drugs at a constant rate over an extended period of time [14], when the rate can be controlled by cross-linking density. Further, the biodegradable platforms can eliminate the need for subsequent removal of the platform. These platforms can be used for local delivery of the therapeutic proteins to the mucosal tissue.

Due to the ability to be processed at a low temperature, high water-solubility, non-toxicity, and biodegradable nature, gelatin has become a widely used collagen-based proteic polymer [15,16,17]. Gelatin-based polymer composites are widely used in many application areas, including electronic devices [18], drug delivery platforms [19], and food packaging [20]. Furthermore, gelatin-based polymers are also used in protein [21] and growth factor delivery [22] platforms. The highly complex structure of the protein with several charged sites should be taken into consideration during the design of a carrier network [23]. As a drug delivery platform, gelatin offers a facile chemical structure with many charged functional side groups. The flexibility in electronic properties of the gelatin-based platforms makes them suitable for a wide range of applications in areas from tissue engineering, to drug delivery systems, and degradable platforms for bioelectronics [24,25,26,27,28,29,30]. For instance, alkaline-processed gelatin has a greater number of carboxyl groups, rendering a negative charge with an isoelectric point around 5.0 [31]; while acidic-processed gelatin has an isoelectric point of 9.0 and positive charge. The multiple points of interaction between the load and gelatin network prevents the dissociation of the load molecule (e.g., protein) readily. As a result, the degradation profile of the carrier polymer network will be the determining factor during drug/protein release.

Gelatin-based platforms’ thermal and mechanical stability can be improved by utilizing various cross-linking methods, as well as control over their degradation under physiological conditions. Physically-initiated cross-linking methods, like UV radiation and dehydrothermal treatment, were found to be inefficient in controlling the degradation of the material [32]. On the other hand, use of glutaraldehyde (GA) has shown to be more efficient, while utilizing cross-linking density to gain control over degradation has been widely documented [33,34,35]. The most common GA-based cross-linking method is mainly based on the addition of a certain amount of GA in the gelatin solution, casting films, and drying them. The toxicity of GA is related with the amount of the released GA. It is shown that the GA released from gelatin films is negligible when cross-linked with less than 2 vol % GA [36]. In a recent study, polyvinyl alcohol (PVA) and gelatin composite hydrogels were studied as IPNs with excellent biocompatibility [37]. Furthermore, in a previous study, we have demonstrated controlled degradation of PVA and gelatin composites [38]. The properties gained from presence of different types of polymers and different degrees of cross-linking in gelatin-based composites will allow for control over water uptake and drug diffusivity.

One specific application of platforms that can provide controlled release is in the field of vaccine delivery. An ideal vaccine should be able to mount a strong immune response and generate long lasting memory, which is often achieved by multiple rounds of vaccination. However, a delivery platform that can provide a slow and constant release might streamline the traditional vaccination method by removing the need for booster doses. In order to further test the ability of our platform to provide controlled release, a HIV-1 gp41-based protein named gp41-HR1-HR2-54K (Figure S1) has been used as a load in this study. This protein is similar to the “gp41-inter” construct described by Frey *et al.* [39]. The domain structure and sequence of this protein presents an extended pre-hairpin intermediate structure of gp41, which is an important target for vaccine design against HIV-1. The HR1 and the HR2 domain of this protein also form a six-helix bundle structure that can be detected by the NC-1 antibody

**Figure 1:**
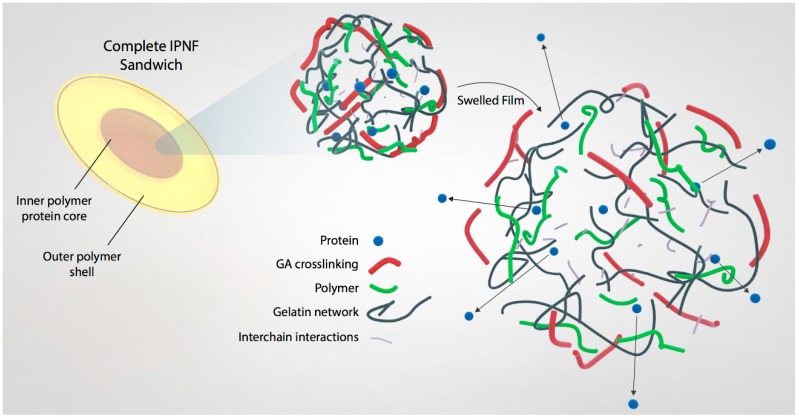
A structural model of the IPNFs pellet. The inner core consists of polymer-gelatin network and the load protein. This inner core is then encapsulated in an outer shell comprising of IPNF only.

The gp41-HR1-HR2-54K protein has an overall positive charge; thus, to maintain the charge, and also to incorporate the protein in the gelatin carrier network through the electrostatic interactions, negative-charged gelatin (type A, alkali-treated) was used. To investigate biodegradability dependence on the chemical structure, we used three biocompatible polymers with gelatin to form three different types of IPN films (IPNFs). Polyethylene glycol (PEG), for its desirable thermal and physical properties; PVA for its desired properties such as hydrophilicity, biodegradability, biocompatibility, and non-toxicity; and hydroxyethylene cellulose (HEC), for its high viscosity and inertness toward the pH of media, were integrated with gelatin and investigated in this study. Control over the release of load protein is achieved via the means of controlling the drug trapping property of gelatin in the IPNFs (Figure 1). For this purpose, two different cross-linkers have been used. Glucose, as a biocompatible inner cross-linker, provided the ECM-like inter-chain interactions. These interactions served as an effective scaffold for the encapsulation of the protein without damaging the secondary structure. Glucose was also selected for its cross-linking and stabilizing effects on gelatin, non-toxicity, and biodegradability [19,40]. It has been observed that glucose has a better cross-linking effect compared to sucrose [22]. Glucose, as a cross-linking agent, provides the inner network bindings while maintaining the protein structure. GA, as an outer shell forming cross-linking agent, served as the main content to control the degradation of the platform in long-term and maintained sustained delivery. GA-based cross-linking is non-specific, which has a potential to damage the loaded biomaterial [35]. This sort of damage would inactivate the therapeutic property of the load. The method offered here actually hides the loaded biomaterial from direct interaction of the cross-linker and maintains its activity.

The significance of the method offered here is the simplicity. It would be possible to design a protein encapsulation media for different biomaterials by using this method and adjusting the mixture composition. The method, which is based on the dipping of the platform in the cross-linking agent solution, provides cross-linked outer shell to the pellet platform for long-term transiency without damaging therapeutic proteins or vaccines, which can be easily applied for the bulk production for such long-term drug delivery platforms. Traditional gelatin-based drug delivery platforms are prepared with mostly toxic and environmentally hazardous chemicals, such as toluene and chloroform [35]. Here, we are offering a new method, which is entirely environmental friendly, simple, and non-toxic.

## 2. Materials and Methods

### 2.1. Materials

Gelatin (Type A) (Prionex, highly-purified), polyethylene glycol (*M_w_*: 20,000 g·mol^−1^), polyvinyl alcohol (*M_w_*: 61,000 g·mol^−1^, 98.0–98.8 mol % hydrolyzed), 2-hydroxyethylene cellulose (*M_w_*: 90,000 g·mol^−1^), glycerol, sucrose, and glutaraldehyde were purchased from Sigma Aldrich (St. Louis, MO, USA). NHS-Rhodamine was purchased from Thermo Scientific, Pierce Biotechnology Inc. (Rockford, IL, USA). LDH cytotoxicity assay (Pierce) and HeLa cell lines were used for cytotoxicity assay. The cell media was DMEM with 10% FBS and 1% Penicillin-Streptomycin.

### 2.2. IPNF Preparation

The films were prepared via solvent casting method. In brief, 5 wt % solution of gelatin in water was prepared with 1.5 wt % glucose and 1.5 wt % glycerol. Glycerol was used as a plasticizer agent. Due to its low molecular weight and hydrophilic nature, glycerol fits well into proteic network and reduces protein-protein interactions by establishing hydrogen bonding with amino acid residues [41,42,43]. To elongate the shelf-life, a combination of plasticizers like glycerol:sorbitol [44] and glycerol:sucrose [45] were studied. To prepare IPNFs, additional to the mixture described above, 0.5 wt % one of PVA [38,46], PEG [47] or HEC [48] was added as shown in Table 1. The resulting solution was than casted on circular templates (8 mm diameter) and left at ambient conditions to dry for 24 h. The dried films were then peeled off (8 mm diameter × 100 µm thickness) and dipped in GA aqueous solutions of different volume ratios (0.1, 0.25, 0.5, and 1 vol %) for 6 h at 4 °C to cross-link. Finally, the cross-linked films were washed with distilled water to remove the excess GA and dried at ambient conditions for 24 h.

### 2.3. Gravimetrical Analysis of Degradation

The degradation rates of polymer films were determined through the measurement of the change in the films’ weight (weight loss) as a function of time. Prior to gravimetrical analysis, the initial film weight of each sample was determined on an analytical balance (Mettler Toledo XS105 Dual Range, Columbus, OH, USA) and then put in 3 mL PBS (1×) buffer at pH 7.4 at 37 °C to determine the degradation in physiological fluid. At specified time intervals (one day; two, three, and four weeks) the samples were removed from the PBS buffer, gently blotted to remove adherent buffer and subsequently weighed to determine the wet weight of the films. The water content of the samples was calculated from the difference between the swelled wet weight of IPNFs and initial weight of dry IPNFs. Relative weight loss was calculated as the difference between the final and initial wet weights with the presumption that the water ratio remained the same throughout the degradation process.

### 2.4. Cytotoxicity Evaluation

Sample films (8 mm diameter × 100 µm thickness) were maintained in cell growth medium (300 µL) at 37 °C. The supernatant medium was collected at specified time intervals (one day; one, two, three, and four weeks) and used for performing LDH cytotoxicity assay as per the manufacturer’s (Pierce/Thermo Scientific; cat #88953) instructions with minor changes. Briefly, cells were seeded at 10,000 cells/well and incubated overnight at 37 °C in a 5% CO_2_ incubator. To test samples, the medium was replaced with collected supernatant from sample films and incubated for an additional 24 h. The same procedure was followed for control samples but using normal growth medium. Supernatants were collected the next day, processed, read, and the percentage cytotoxicity was calculated as per the manufacturer’s instructions.

### 2.5. Preparation of Protein Loaded Pellets

The gp41-HR1-HR2-54K protein was tagged with NHS-Rhodamine as described in the manufacturer’s protocol. The tagged protein was mixed with 5 wt % gelatin solution (prepared as described before with glycerol and glucose) at a weight ratio of 1:100 (weight ratios of 1:50 and 1:200 were also tested for optimization of protein loading). Then, this mixture was dropped on dried IPNF (12 mm diameter × 100 µm thickness) and left to partially dry at room temperature for 24 h. Then, an identical film (12 mm diameter × 100 µm thickness) was used to cover the partially-dried protein droplet and left to fully dry for 24 h. The IPNF coating was then cross-linked by exposure to GA solutions at different concentrations (0.1, 0.25, 0.5, and 1 v/v % GA). Cross-linking was performed at 4 °C using pre-chilled GA solution for 6 h. After cross-linking, the films were washed with abundant distilled water to remove the GA residue and dried at ambient conditions for 24 h, (Figure S2).

### 2.6. In Vitro Release of Protein

Protein loaded pellets of different IPNF samples (12 mm diameter × 200 µm initial thickness) were maintained in PBS (1×) at 37 °C. The supernatant was collected at specified time intervals (one day; one, two, three, and four weeks) and replaced with PBS solution to keep the volume constant. At each reading the amount of protein released in the supernatant was measured using spectrofluorometry (SpectraMax M2e Microplate Reader, Molecular Devices, Sunnyvale, CA, USA) and added to the previously-measured protein amount to get the cumulative release curve.

### 2.7. ELISA Test

ELISA was performed as previously described [49] with minor modifications. Labeled proteins (both unloaded and released) were coated onto 96-well Nunc-Immuno plates overnight at 4 °C at 30 ng per well using an antigen coating buffer (15 mM Na_2_CO_3_, 35 mM NaHCO_3_, and 3 mM NaN_3_, pH 9.6). Uncoated surfaces were blocked with 200 μL of a blocking buffer (PBS, pH 7.4, containing 2.5% skim milk, and 25% Fetal Bovine Serum) for 1 h at 37 °C. Wells were subsequently washed five times with a wash buffer (PBS containing 0.1% Tween 20) using a Biotek automated plate washer. As a primary antibody, NC-1 was serially diluted in a blocking buffer (1000 ng/mL starting concentration), and 100 μL was added to each well. Plates were then incubated for 2 h at 37 °C. Wells were washed ten times, and plates were further incubated for 1 h at 37 °C with a secondary antibody (horseradish peroxidase (HRP)-conjugated goat anti-human IgG (H + L); Pierce) at 1:3000-fold dilution in the blocking buffer. Wells were washed again ten times, and the HRP reaction was initiated by adding 100 μL TMB HRP-substrate (Bio-Rad, Hercules, CA, USA). The reaction was stopped after 10 min by adding 50 μL of 2 N H_2_SO_4_. Plates were read on a microplate reader (Versamax by Molecular Devices, Sunnyvale, CA, USA) at 450 nm. All assays were done in duplicate.

## 3. Results and Discussion

The release of proteins from the IPNFs depends on their dissolution rates. It is expected that IPNFs with higher GA cross-link density would dissolve slower and, hence, be more suitable for long-term release of proteins as opposed to IPNFs with lower GA cross-link density. However, a harsh cross-linker like GA forms strong covalent bonds between the host matrix and load (drug or protein). Such chemical bonds decrease the effectiveness of the load by changing their chemical nature [50,51,52]. To overcome this problem, we designed a unique method for fabrication of core-shell IPNFs, as presented in Figure 1. In this method, we mixed the protein with gelatin-based IPNF and encapsulated this mixture in between two protein-free, polymer-enhanced IPNF to form an IPNF pellet. We then dipped this pellet into the GA solutions at a low temperature to form an outer cross-linked shell (shown in red in Figure 1). By this method, we prevented the direct contact of GA with proteins, which could result in denaturation and loss of therapeutic activity or immunogenicity. At the same time, we benefit from the extra cellular matrix-like structure of the gelatin-based film to load and retain the proteins for an extended time without losing the effect.

### 3.1. Degradation Profiles of Polymer Composites

**Figure 2:**
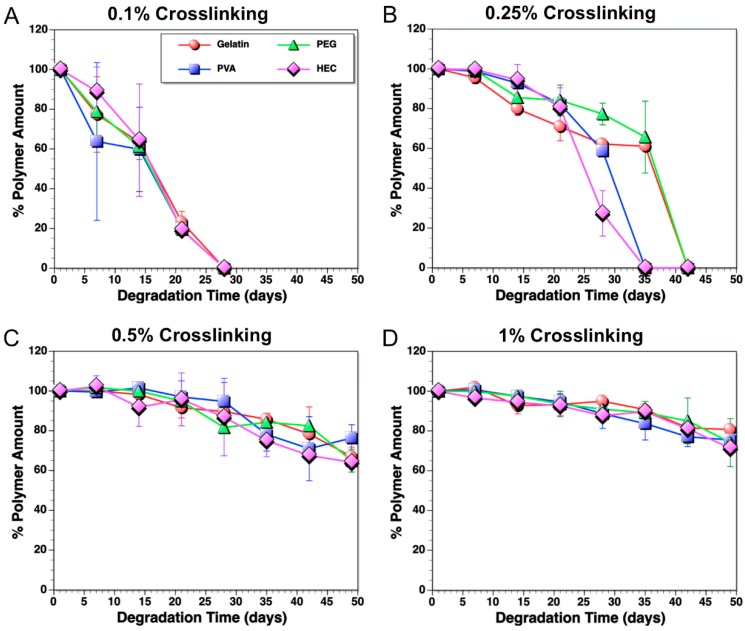
Degradation of polymers in PBS solution (pH 7.4) at 37 °C, which were cross-linked with different GA volume concentrations: (**A**) 0.1 vol %; (**B**) 0.25 vol %; (**C**) 0.5 vol %; and (**D**) 1 vol %.

To investigate and establish a correlation between the extent of cross-linking based on the concentration of GA and degradation time, the first set of experiments was performed on unloaded IPNFs. The degradation of the IPNFs was investigated with one-layer films (Figure 2). It has been previously shown that in the hydrogel systems, the process of swelling and dissolution (or degradation) rates compete against each other [53]. The hydrogels showed a maximum weight increase due to swelling with the solvent in the first 24 h. Hence, the first day weight was taken as the 100% swelled film weight. It was used as the initial wet weight for the further calculations. The weight ratio between the water content and IPNF was calculated from the difference between the initial wet film and the dry film before solvent immersion; this weight was considered to be constant throughout the degradation process. To distinguish between membrane loss and water loss, at each measurement, the swelled film was weighted and the change was multiplied by the water content ratio.

As shown in Figure 2, samples cross-linked in higher GA concentration dissolved slower compared to ones cross-linked in lower GA concentration. The observation of degradation of the IPNFs was consistent with our hypothesis. IPNFs cross-linked with 0.1 vol % GA dissolved completely in 3 weeks (Figure 2A). The cross-linking with 0.25 vol % GA increased the degradation time of the IPNFs to 5–6 weeks, while the ones cross-linked with 0.5 vol % GA remained even after 8 weeks. Uncross-linked IPNFs, even with the presence of glucose in the network, dissolved in 10 min in PBS solution (pH 7.4) at 37 °C (data not shown).

The degradation of the IPNFs started after the swelling process reached a saturated state, as can be observed from the constant weight values (Figure 2) [54]. The solubility of cross-linked IPNFs is less likely to start with diffusion of water into the film network. The water intake by the IPNFs reached a maximum at the end of the first 24 h (hence, this weight is taken as the initial wet weight). At this maximum volume, water remained in the hydrogel for a period of time, depending on the cross-linking ratio. The water pressure in the swelled network would disturb the polymer chains and cause dislocations on the very outer surface of the network. The initiation of dislocations triggers the overall degradation immediately. During this process, the presence of water-soluble biopolymers may affect the swelling, degradation, and further interaction with the loaded protein. The biopolymers may show different interactions with different types of proteins and drugs loadings. These interactions are likely to be a result of different electronic charge, chemical structure, and polarity of different proteins and drugs. These parameters will be the advantages for drug design and bioenvironment-specific platforms.

The swelling rate of the IPNFs was also studied (data not shown). Under identical conditions, PVA- and HEC-based IPNFs swelled significantly more than PEG IPNFs and gelatin films. The difference of the chemical structure and the hydrophobicity of the polymers might be the reason of this observation. However, this difference in swelling did not translate to differences in complete degradation times, especially in case of 0.1 vol % and 0.5 vol % GA cross-linked IPNFs. Interestingly, PVA and HEC IPNFs cross-linked using 0.25 vol % GA degraded over a period of time almost one week shorter than their PEG and gelatin counterparts. This could be due to the differences in the chemical structures and functional groups of the polymers. Although, the more hydrophilic compounds are expected to be more water-soluble, stronger interactions of the functional groups of gelatin may cause less degradation.

The swelling ratio of the polymer is simply defined as the maximum amount of water remaining in the polymer network before the entanglement of the polymer molecules start. Entanglement is the displacement of a polymer chain in a network and occurs as a result of loss of non-covalent interactions in between the polymer chains and enlargement of the free space to move around the molecule. The absorption of water molecules between the polymer chains starts the swelling of the polymer by breaking the intermolecular H-bonds. The enlargement of the space between the polymer chains decreases the electrostatic interactions and gives higher mobility to polymer chains. The more hydrophilic polymer chains would be surrounded by more water molecules, and would degrade faster. On the other hand, the less hydrophilic polymer chains would have more hydrophobic interactions, which would even get stronger with the presence of water, and degrade slower.

### 3.2. Cytotoxicity Assay

GA is commonly used as a cross-linker agent, specifically for collagen-based materials. The cytotoxic effect of GA has been studied in detail and has been shown that above specific concentrations (2 vol %) it is cytotoxic for many different tissues in the body [55]. As mentioned in the experimental section, all the samples were washed thorough after the cross-linking. Despite washing our IPNFs thoroughly after cross-linking, there is a slight possibility that residual GA might still be present on the exterior surface of the IPNFs. The maximum GA concentration of the cross-linking condition was 1 vol %; thus, the cytotoxicity studies were performed using different types of IPNFs cross-linked with 1 vol % GA. Briefly, cross-linked IPNFs were incubated in cell culture media for different durations of time (Figure S3). This culture media was then collected and used for cytotoxicity testing. It was observed that more than 90% of the cells survived the test and were alive at the end of a three-week assay period (Figure S2). These *in vitro* results show that IPNFs prepared using our method are safe to use for biological applications.

### 3.3. Determination of Protein Loading Capacity of IPNFs

**Figure 3:**
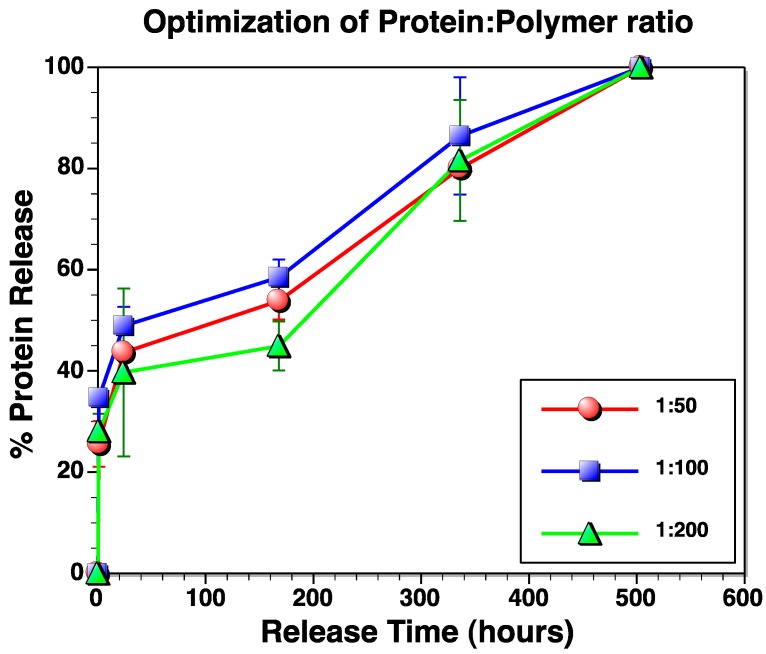
Optimization of protein loading. All polymer films were cross-linked with 0.25 vol % GA. Release rates were similar even in the case of different protein to polymer loading ratios.

As a result of different interactions of the functional groups, cross-linking ratios, electrostatic interactions, and hydrophilicity, the maximum loading capacity of different IPNFs is expected to be different. In order to determine the maximum protein loading capacity of IPNFs, different amounts of NHS-Rhodamine-labeled protein was loaded in gelatin films (without additives). All samples were prepared and loaded directly by adding the protein to the inner gelatin matrix. Three different loading ratios were tested based on mass equivalent ratios; 1:50, 1:100, and 1:200. The ratios calculated by keeping the gelatin amount constant and only varying the protein amount to avoid any potential discrepancy. All of the IPNFs were cross-linked in 0.25 vol % GA, after encapsulation of the protein-network mixture. At the end of the release tests, we observed that there was no significant difference in the protein release rates between different loading ratios at 0.25 vol % GA cross-linking concentration (Figure 3). The similarity of the release ratio is mainly due to the electrostatic interactions between the loaded protein and the gelatin network. The gelatin network might be able to hold the protein in the network to some extent with the help of the glucose cross-linker. Since the initial burst release was also similar between different loading ratios, the gelatin films were not oversaturated even at high protein amounts. Based on these results, we decided to use a ratio of 1:100 for future characterization with different IPNFs.

### 3.4. Protein Release Kinetics

**Figure 4:**
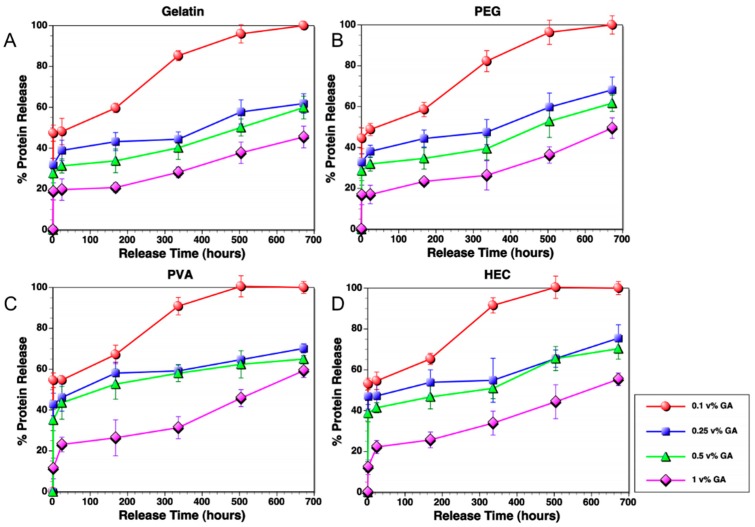
Release of protein from different IPNFs (**A**) gelatin film pellet; (**B**) PEG-enhanced IPNF; (**C**) HEC-enhanced IPNF; and (**D**) PVA-enhanced IPNF in PBS solution (pH 7.4) at 37 °C after cross-linking with different GA volume concentrations (red: 0.1 vol % GA, blue: 0.25 vol % GA, green: 0.5 vol % GA, purple: 1 vol % GA).

As described above, the variety of GA concentration results in different cross-linking densities and, thus, different degradation times. To identify the protein release kinetics of differently cross-linked IPNFs, we used four different GA concentrations (0.1 vol %, 0.25 vol %, 0.5 vol % and 1 vol %), exactly as used for single layer IPNF cross-linking (Figure 4). Regardless of the type of polymer composites used in IPNFs, the overall pattern of protein release kinetics was similar (*i.e.*, rapid release within one or two days followed by more gradual, slow release over time). The release rates decreased significantly when the higher concentration of GA was used for cross-linking (0.1 vol % *vs.* 1 vol %). However, no significant difference was observed between release rates of IPNFs cross-linked with 0.25 vol % and 0.5 vol % GA suggesting that there may not be significant difference in the cross-linking densities between these IPNFs. 0.25 vol % GA concentration was found to be sufficient for the formation of a cross-linked outer-shell on IPNFs. The outer-shell, to some extent, allows the internalization of water molecules, swelling, which opens up the intermolecular free space and this space continues to increase throughout the degradation process. Although the protein molecules are significantly larger than water molecules, and are also held by the electrostatic interactions of the gelatin network, they eventually diffuse out through the opened up space, which reaches the sufficient size for protein release, and exhibit a gradual release behavior. On the other hand, at higher concentration (1 vol %) GA might penetrates further into the network and result in deeper cross-linking. The deeper cross-linking requires more time to internalize water molecules and reach the sufficient size for protein release; as a result, the release behavior exhibits a different behavior and occurs over a longer period of time.

The release behavior of IPNFs here showed a biphasic release profile [56]. The initial and burst release of the drug is the result of diffusion of protein from the surface through the swelled and distracted outer shell of the IPNF. This diffusion continues by the depletion of free protein located in the periphery of the inner pellet. Then the release slows down and a plateau forms. The protein encapsulated in the gelatin network releases by the degradation of the matrix and the second phase of release appears. Obviously, diffusion of the protein, swelling, and degradation of the IPNF are all playing a role and make the release behavior of the film complex.

The release data clearly shows that protein release from these IPNFs can be manipulated by changing the extent of cross-linking via GA treatment. This property can be especially useful for drug delivery and other applications, in which the release period is essential. However, it must be noted that the exact release rates are expected to be influenced by the size and charge of the load molecules; thus, load-specific optimization is likely required for each case.

### 3.5. Determination of Protein Structure upon Release

**Figure 5:**
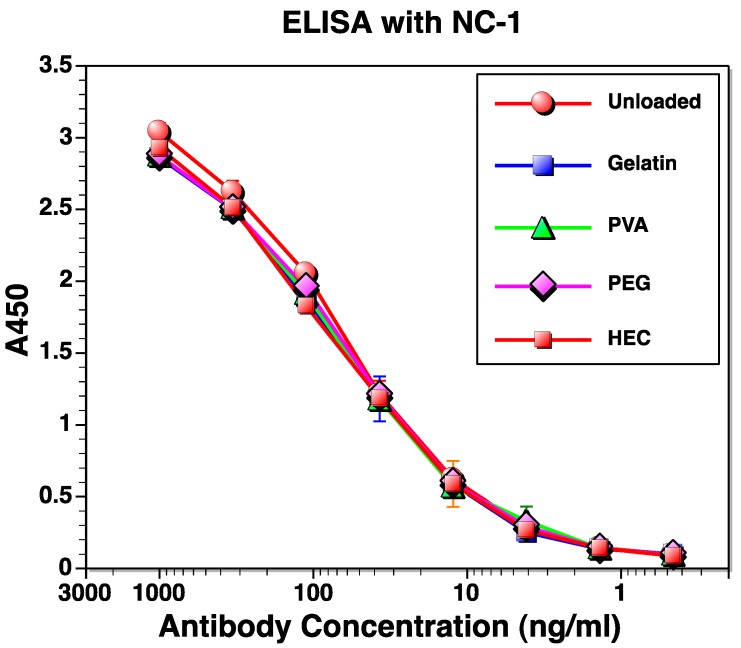
Structural conservation of released protein from different IPNFs. Labeled protein released from IPNFs cross-linked with 1 vol % GA was compared with unloaded labeled protein using ELISA with a NC-1 monoclonal antibody.

Maintaining their structural integrity upon release is a major criterion for biomolecules to allow retention of their functionality. The protein used in this study is a gp41-based HIV-1 envelope protein that has a unique six-helix bundle conformation. This conformation can be readily detected by mouse monoclonal antibody NC-1 using traditional ELISA [57]. We examined and compared the binding of this antibody to the released proteins and the unloaded NHS-labeled protein to confirm the structural integrity of the released proteins. As shown in Figure 5, the released proteins from different IPNFs showed similar binding efficiency as the unloaded protein. This suggests that the protein structure is conserved during the loading and release processes.

## 4. Conclusions

Here, a very simple method of preparing biodegradable gelatin-based platforms for long-term release of proteins has been described. Basically, the method is dipping the protein-encapsulated pellets in the cross-linking agent solution. The adjustment of the polymer content and inner cross-linkers of the platform provide the convenient medium for large variation of proteins, growth factors, and other therapeutic materials, which are susceptible to the environmental conditions. The long-term, slow release of the protein was achieved by the adjustment to the concentration of the cross-linking agent in the solvent and also the duration of the dipping. The method is simple enough to apply to bulk production. The designed IPNF platform, the pellet, is designed to mimic the natural extracellular network.

Our method for generation of GA cross-linked IPNFs is successful in its ability to deliver controlled release while preventing chemical denaturation of the loaded protein. We have further shown that this method can be extended to a variety of polymers that can be used for generation of IPNFs designed for different applications. However, one caveat of this current study is the size of the IPNFs is somewhat large. While these IPNFs can be used as a patch for topical delivery of proteins, it is not suited for delivery of proteins through intravenous, subcutaneous, intramuscular, or other invasive routes. With regards to this, several groups have described the generation and testing of polymer-based nanoparticles [52,58]. We believe that the concepts demonstrated in this paper when extended to such polymeric nanoparticles might be able to significantly prolong delivery without compromising the property of the delivered protein.

## Figures and Tables

**Table 1:** Chemical structure of IPNFs.

Sample	Constituents
GEL	5 wt % gelatin
GEL-PVA	5 wt % gelatin + 0.5 wt % PVA
GEL-PEG	5 wt % gelatin + 0.5 wt % PEG
GEL-HEC	5 wt % gelatin + 0.5 wt % HEC

## Supplementary Materials

The following are available online at www.mdpi.com/1996-1944/9/5/321/s1.
