# Supplementary material for: Transient Biocompatible Polymeric Platforms for Long-Term Controlled Release of Therapeutic Proteins and Vaccines
Source: Materials (Basel). 2016 Apr 28;9(5):321. doi: 10.3390/ma9050321 (PMC5441878; doi:10.3390/ma9050321)
Supplement: Supplementary file 1 [file materials-09-00321-s001.pdf]

Supplementary Materials: Advanced Gelatin-Based Polymer Platform for Long-Term Release of Therapeutic Proteins or Vaccines

Handan Acar, Saikat Banerjee, Heliang Shi, Reihaneh Jamshidi, Nastaran Hashemi, Michael W. Cho and Reza Montazami

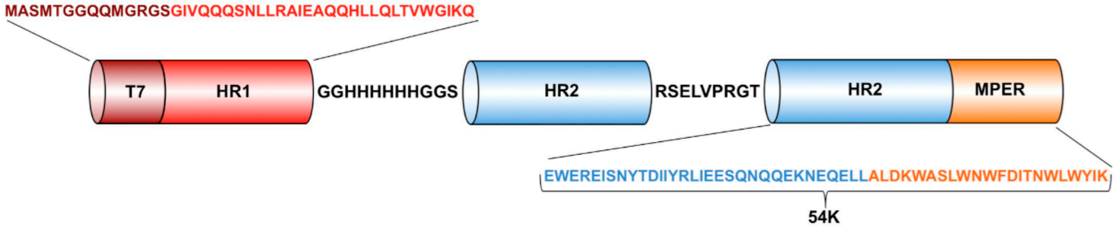

Figure S1. Domain structure and sequence of gp41-HR1-HR2-54K. The protein contains a T7 expression tag, HR1 domain, 6xHis linker, HR2 domain, thrombin cleavage linker, HR2 domain, and MPER domain.

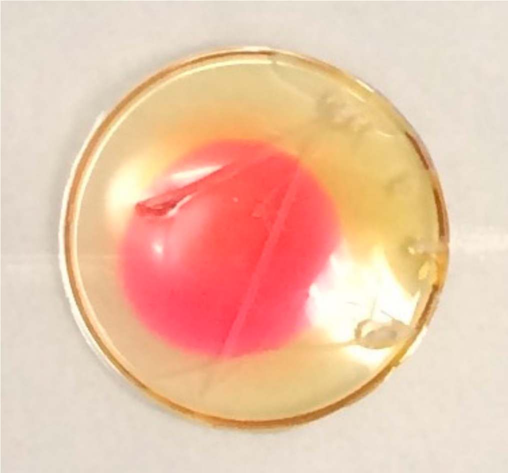

Figure S2. Photograph of an IPNF, rhodamine-labeled protein (pink core) is encapsulated in a gelatin-based pellet.

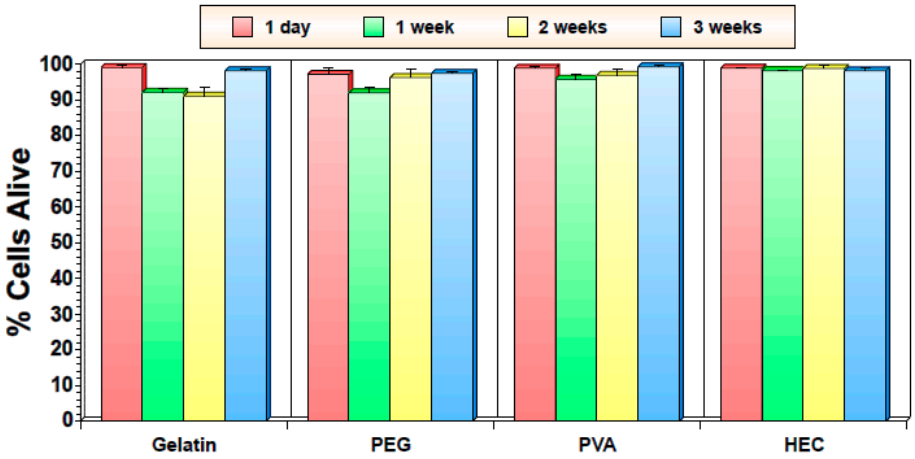

Figure S3. Cytotoxicity of IPNFs. The different IPNFs showed little to no cytotoxicity *in vitro*, even after three weeks of incubation.
